# A Novel Microtubule-Disrupting Agent Induces Endoplasmic Reticular Stress-Mediated Cell Death in Human Hepatocellular Carcinoma Cells

**DOI:** 10.1371/journal.pone.0136340

**Published:** 2015-09-10

**Authors:** Chun-Te Ho, Yu-Jia Chang, Li-Xi Yang, Po-Li Wei, Tsan-Zon Liu, Jun-Jen Liu

**Affiliations:** 1 Graduate Institute of Medical Sciences, College of Medicine, Taipei Medical University, Taipei, Taiwan; 2 School of Medical Laboratory Science and Biotechnology, Taipei Medical University, Taipei, Taiwan; 3 Translational Research Laboratory, Cancer Center, Taipei Medical University and Hospital, Taipei, Taiwan; 4 Department of Surgery, Taipei Medical University and Hospital, Taipei, Taiwan; 5 Radiobiology Laboratory, California Pacific Medical Center Research Institute, San Francisco, CA, United States of America; 6 St. Mary’s Medical Center, San Francisco, CA, United States of America; Institute of Biochemistry and Biotechnology, TAIWAN

## Abstract

Here, we present evidence of a novel microtubule-disrupting agent, N-deacetyl-N-(chromone-2-carbonyl)-thiocolchicine (TCD), exhibiting potent antitumor activity (with IC_50_ values in the nanomolar range) against hepatocellular carcinoma cell lines. Cell cycle analysis revealed that TCD induced G_2_/M cell-cycle arrest in a dose- and time-dependent manner in both Hep-J5 and Mahlavu HCC cell lines. TCD also induced a decrease in mitochondrial membrane potential (ΔΨm) and caused DNA damage. Mechanistically, TCD activated protein kinase RNA-like endoplasmic reticular kinase and several transcription factors, including activating transcription factor (ATF) 6, ATF4, ATF3, and the CCAAT-enhancer binding protein homologous protein. These data clearly demonstrate that the antitumor activity of TCD is mechanistically linked to its capacity to trigger both intrinsic and extrinsic apoptotic cell death via endoplasmic reticular stress pathway. The potent antitumor activity of TCD was similarly demonstrated in a hepatocellular carcinoma xenograft model, where 5 and 10 mg/kg doses of TCD significantly arrested Hep-J5 and Mahlavu tumor growth. Our finding suggests that TCD is a promising therapeutic agent against hepatocellular carcinoma; further translational assessment of its clinical usage is warranted.

## Introduction

Hepatocellular carcinoma (HCC) is the fifth most common cancer worldwide and the second most common cause of cancer-related deaths annually [1]. HCC is predominantly found in developing countries within Asia and Africa, and it also has a high occurrence rate in Japan, Western Europe, and the United States [2]. Current curative treatments, such as surgical resection and liver transplantation, are not highly effective and can only be applied to early-stage HCCs [3,4]. Only 10–15% of patients receive curative surgery. The majority of HCC patients undergo systemic chemotherapies or supportive therapies, and most chemotherapeutic agents show poor effectiveness with limited improvement in patient survival rates. The multi-kinase inhibitor, sorafenib, was approved as a first-line therapy for patients with unresectable HCCs [4, 5]. In a randomized phase III study, overall median survival rates slightly increased from 4.2 months in the placebo group to 6.5 months in the sorafenib treatment group [5]. However, adverse side effects have limited the drug’s overall usefulness, and there remains a critical need to find more effective therapeutic alternatives.

Microtubules are filamentous polymers that are formed by α-tubulin and β-tubulin heterodimers. The dynamic microtubule structure maintains cell polarity and motility, provides a scaffold for cellular protein and organelle trafficking, and plays a critical role in mitosis [6, 7]. Thus, microtubules act as cell-shape regulators, conveying intracellular signals from growth factors, the extracellular matrix, and cell-cell interactions [6]. Therefore, microtubules are an important target for inducing mitotic arrest and cell death in cancer treatment [8–10].

Three distinct classes of microtubule-targeting agents (MTAs), *Vinca* alkaloids, taxanes, and colchicines, are well studied [11, 12]. *Vinca* alkaloids, including vinblastine and vinorelbine, form a group of microtubule-destabilizing agents used to treat hematological malignancies as well as some solid cancers [12, 13]. Taxanes, such as paclitaxel and docetaxel, are microtubule-stabilizing agents that are clinically useful in treating solid cancers, like breast, lung, ovarian, liver, head, and neck carcinomas [11, 14]. The third group of microtubule-disrupting compounds comprises colchicine and colchicine derivatives, such as thiocolchicine [15]. These compounds are similar to *Vinca* alkaloids, but their binding site and microtubule depolymerization mechanism are different [8, 16].

Colchicine, isolated from *Colchicum autumnale* and *Gloriosa superba*, is a well-known natural product [17]. Colchicine is used as an antitumor agent, and is also used to treat gout. However, owing to their high toxicities, colchicine and thiocolchicine are still in preclinical trials to determine their potential anti-tumor activity. To reduce the toxicity without reducing anti-cancer effects, colchicine analogs were developed [18, 19]. Some colchicine derivatives were shown to inhibit endothelial cell proliferation, which would arrest existing tumor vasculature [19]. Another study has shown that perioperative administration of colchicine inhibited the surgical activation of malignant cells, thus enhancing tumor-free survival in vivo [20]. However, it is necessary to further define the effects of colchicine derivatives in different cancer types and their underlying mechanisms.

MTAs cause mitotic arrest that leads to cell apoptosis, but this mechanism may not be applicable in patients whose tumor cells divide every 30−60 days [21]. Therefore, aside from primary anti-mitotic toxicity, evidence that MTAs induce cell death through other signaling mechanisms may have clinical implications in cancer therapy. Previous studies showed that both the construction and assembly of the endoplasmic reticular (ER) network [22] and stress-induced misfolded protein aggregation [23] are related to the microtubule network. This implied that MTAs might be involved in cell stress and ER stress signaling. Thus, their crucial involvement in mitosis and the disturbance of stress-related signal transduction make these signaling proteins targets for MTAs [24, 25]. However, the mechanism underlying this association is unclear.

Here, we report a new thiocolchicine derivative, N-deacetyl-N-(chromone-2-carbonyl)-thiocolchicine (TCD) (S1 Fig), as an anticancer agent that acts in a manner different from that of classical tubulin inhibitors. Because colchicine was shown to have therapeutic efficacy in HCC [26], TCD was applied to different HCC cell lines to evaluate its potency as an anticancer agent. Our results showed that TCD arrested mitotic cells in the G_2_/M phase by depolymerizing microtubules in the HCC cell lines Mahlavu and Hep-J5. TCD mediated HCC cell apoptosis through caspases-dependent pathway and the induction of ER stress. These antitumor activities were also extended to an in vivo animal study, where TCD administration significantly inhibited HCC growth.

## Materials and Methods

### Cell lines and reagents

TCD (C_30_H_27_NO_7_S), an odorless, yellow crystal powder, is a novel colchicine derivative; it was purchased from PUMC Pharmaceutical Co., Ltd. (Bejing, China) for this study. This water-insoluble compound was dissolved in DMSO (Sigma-Aldrich, St. Louis, MO, USA) to be used as a stock solution for further in vitro studies. Three human hepatoma cell lines, Hep-J5, Mahlavu, and SK-Hep-1, were kindly provided by Professor Kwang-Huei Lin [26, 27], among which SK-Hep-1 was originally purchased from the American Type Culture Collection, VA, USA [26], and J5 and Mahlavu were originally obtained from Dr. C.S Yang [28], National Taiwan University, and Dr. C. P. Hu [27], Veterans General Hospital, Taiwan, respectively. Mahlavu was originally established from a female hepatoma patient in 1972 and has been in continuous culture since then [29]. Huh7 was purchased from the Japanese Collection of Research Bioresources Cell Bank, Osaka, Japan. Hep-G2 and Hep-3B were purchased from the Bioresource Collection and Research Center, Hsinchu, Taiwan. Dulbecco’s modified Eagle medium (DMEM) and fetal bovine serum (FBS) were obtained from Sigma-Aldrich and Biological Industries (South Logan, UT, USA), respectively. All other chemicals were purchased from Sigma-Aldrich.

### Cell culture and treatment

All cells were cultured in DMEM containing 10% FBS, and were incubated under a humidified atmosphere with 5% CO_2_ at 37°C. To treat hepatoma cells, different concentrations of TCD or colchicine were prepared in culture medium. Control cells were incubated with DMSO, at a concentration equivalent to that for cells receiving the test compound solution. For dose-dependent TCD treatments, 2×10^6^ cells were plated in 10-cm dishes. After cells adhered to the culture dish, the medium was removed, and cells were treated with 5, 10, or 20 nM TCD for 24 h. For time-dependent treatment, 5×10^5^ cells were seeded on 10-cm dishes and were allowed to adhere. The cells were then treated with 10 nM TCD for 9–72 h.

### Cell proliferation assay

Cell proliferation was measured using the sulforhodamine B (SRB) method. After incubation with different concentrations of TCD, cells were fixed with trichloroacetic acid for 1 h at 4°C and then stained with 50 μl SRB solution (0.4% in 1% acetic acid; w/v) for 10–20 min at room temperature. After staining, the SRB solution was removed, and test wells were washed twice with 1% acetic acid before air-drying. Subsequently, bound SRB was solubilized with 200 μl of an unbuffered Tris-based solution (20 mM). The absorbance was measured by a spectrophotometer at 540 nm.

### Immunocytochemistry

After TCD treatment, cells were washed with PBS and fixed in 3.7% formaldehyde. Cells were then permeabilized for 30 min with phosphate-buffered saline with 0.5% Triton X-100 and blocked with PBST (PBS with 0.1% Tween-20) containing 1% FBS for 30 min. Cells were incubated with a primary monoclonal antibody against α-tubulin (1:1000 dilution in PBST; Sigma-Aldrich) overnight at 4°C or 60 min at room temperature. After incubation, cells were washed three times with PBST and were incubated with a Cy3-conjugated secondary antibody (1:2000 dilution in PBST; Jackson ImmunoResearch, West Grove, PA, USA) in the dark. Cell nuclei were stained with DAPI. Images were captured with a fluorescent microscope.

### Cell-cycle analysis

After TCD treatment, cells were washed with PBS and fixed with a PBS-methanol solution (1:2, v/v) overnight at 4°C. Fixed cells were washed twice with PBS solution and stained with 48 μg/mL propidium iodide (PI) containing DNase-free RNase-A (48 μg/mL) in the dark for 20 min at room temperature. The DNA content was analyzed by flow cytometry (BD FACSCalibur, BD Biosciences, San Jose, CA, USA). For each analysis, a minimum of 10^4^ cells were counted and gated, and the percentage of cells in each phase was calculated using ModFit software.

### Cell death analysis by TUNEL assay

Mahlavu and Hep-J5 cells were treated with 10 nM TCD for 24 or 48 h. After TCD treatment, cells were washed with PBS and fixed with 2% paraformaldehyde overnight at 4°C. Apoptotic cell death, characterized by DNA fragmentation, was analyzed using an Apo-BrdU in situ DNA fragmentation assay kit (Promega, Madison, WI, USA). Briefly, fluorescein-12-dUTP was incorporated into fragmented DNA within apoptotic cells at 3′-OH ends using the terminal deoxynucleotidyl transferase enzyme (TUNEL assay) and then quantitated by flowcytometry (BD FACSCalibur; BD Biosciences).

### Mitochondrial membrane potential (MMP) assay

To measure the cellular MMP (Δψm), cells were incubated with 5 μM rhodamine 123 for 30 min after drug treatment. Cells were then washed twice with PBS and trypsinized. Detached cells were centrifuged, re-suspended in PBS, and measured for fluorescence intensity. Changes in the MMP were measured using a FACSCalibur flowcytometer (BD Biosciences) and analyzed using CellQuest software.

### GRP78-knockdown HCC cell generation

GRP78-knockdown Hep-J5 cells were kindly provided by Dr. Yu-Jia Chang [27]. Briefly, small interfering (si) RNA was used to knockdown human GRP78 mRNA. The target sequence for human GRP78 was 5′-AAGGTTACCCATGCAGTTGTT-3′. The control, scrambled siRNA sequence was 5′-AAGGTGGTTGTTTTGTTCACT-3′. GRP78 and scrambled siRNAs were inserted into the pSUPERIOR vector and transfected into Hep-J5 cells. Successfully transfected cells were selected by antibiotic resistance.

### Western blot analysis

After TCD treatment, adherent cells were scraped into 3 ml cold PBS and pelletized by centrifugation, and then proteins were extracted using Total Lysis Buffer (Bioman Scientific Co., Ltd., Taipei, Taiwan) containing protease and phosphatase inhibitors. Equal amounts of protein lysates were resolved using sodium dodecylsulfate–polyacrylamide gel electrophoresis (SDS-PAGE) and electrophoretically transferred to polyvinylidene difluoride (PVDF) membranes. The blots were probed with anti-human primary antibodies, including GRP78, GRP94, activating transcription factor (ATF) 6, ATF4, ATF3, p-p38, CHOP (Santa Cruz Biotechnology, Santa Cruz, CA, USA), anti-PARP-1, anti-procaspase-9 (Epitomics, Burlingame, CA, USA), anti-procaspase-8, anti-procaspase-3 (Millipore, Darmstadt, Germany), and anti-actin (Sigma-Aldrich). Detection was performed using enhanced chemiluminescence (ECL; Millipore) after incubation with a horseradish peroxidase (HRP)-conjugated secondary antibody. Actin was used as the loading control.

### Animal models for the therapeutic study

BALB/c-nu mice (6–8 weeks old and weighing 17–20 g) were purchased from the National Laboratory Animal Center (Taipei, Taiwan). On day 0, mice were subcutaneously injected with 1×10^6^ Mahlavu or Hep-J5 tumor cells. The Mahlavu and Hep-J5 administered groups comprised 60 mice in total, which were divided into 6 groups (3 groups for each tumor cell type). When the mean tumor diameter reached 5 mm, the 3 groups administered Mahlavu or Hep-J5 were further randomly subdivided into 10 mice per group to receive the respective treatment. Experimental mice were treated with formulated TCD at a dose of 5 or 10 mg/kg by tail-vein injection twice a week for 2 weeks. Control mice were treated with an equal volume of normal saline. The tumor size was measured with calipers and calculated using the formula: *a × b* ^*2*^ */2*, where *a* is the length and *b* is the width in millimeters. Tumor size and body weight change were monitored every 3 days after the first drug administration. Test mice were sacrificed after the tumor size reached 3000 and 2500 mm^3^ for Hep-J5 and Mahlavu mice groups, respectively. The animals were euthanized by carbon dioxide anesthesia. To assess treatment-related toxicity, animals were also weighed twice weekly. This study was approved by the Institutional Animal Care and Use Committee (IACUC) of Taipei Medical University. All procedures were performed according to guidelines of IACUC and all efforts were made to minimize animal suffering and the number of animals used. Statistical analysis of tumor volumes between control and TCD-treated mice was performed using a Student’s *t-*test.

### Statistical analysis

Data are expressed as the mean ± standard deviation (SD) from three independent experiments. A paired Student’s *t*-test was used to determine differences between control and treatment groups (Sigma plot 8.0 software). A *p-*value <0.05 was considered statistically significant.

## Results

### TCD exerts potent antiproliferative effects on HCC cells

The SRB assay was used to evaluate the antiproliferative activity of TCD in seven human HCC cell lines (S2 Fig). Table 1 shows IC_50_ values of TCD in all HCC cell lines tested. All HCC cells were sensitive to TCD treatment with IC_50_ values ranging from 2–50 nM. Remarkably, Hep-J5 and Mahlavu cells showed higher responses to TCD, with IC_50_ values of approximately 10 nM. We also found that compared to TCD treatment (IC_50_<50 nM), higher IC_50_ values (of around 190 nM) of colchicine were obtained in the well-differentiated (Huh7) and p53-null (Hep-3B) HCC cell lines. These results demonstrated that TCD had a potent antiproliferative effect on HCC cells.

**Table 1:** Half-maximal inhibitory concentration (IC_50_) values of TCD and colchicine, indicating their cytotoxic effects in hepatocellular carcinoma (HCC) cell lines.

Differentiation	HCC cell line	TCD IC_50_ (nM)	Colchicine IC_50_ (nM)
**Good**	Huh7	14.7 ± 11.3	~190
	Hep G2	27.0 ± 3.1	~12
	Hep 3B (p53 mutant)	17.9 ± 13.0	~190
	Hep J5	7.7 ± 4.9	~10
	Mahlavu (p53 mutant)	7.1 ± 1.5	~15
**Poor**	SK-Hep-1	10.8 ± 1.6	~16

### TCD induced microtubule network disruption and G_2_/M cell cycle arrest

**Fig 1:**
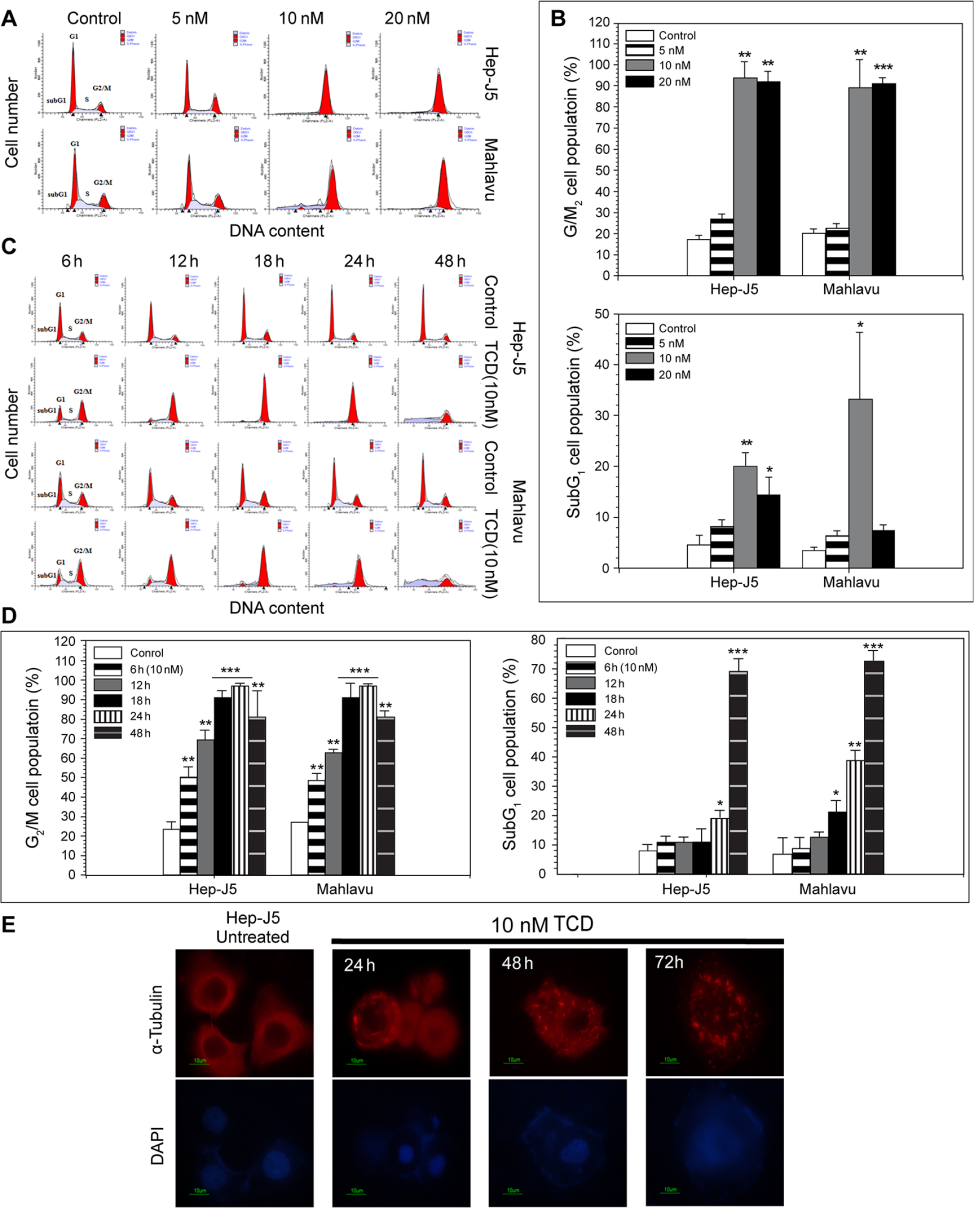
TCD induces G_2_/M phase arrest and disrupts the cellular microtubule network in hepatocellular carcinoma (HCC). (A) The dose effect of TCD on cell-cycle progression was evaluated in Mahlavu and Hep-J5 cells. Cells were treated with TCD for 24 h and analyzed for PI-stained DNA content by flowcytometry. (B) The G_2_/M and subG_1_ populations were analyzed by ModFit software and showed increase in the 10 or 20 nM TCD-treated group (*p < 0.05; **p < 0.01; **p < 0.001). (C) The time effect of TCD (10 nM) on cell-cycle progression was evaluated in Mahlavu and Hep-J5 cells. The G_2_/M and subG_1_ populations showed increase in 24 and 48 h in the TCD-treated group (D). Apoptotic cell death is represented by the sub-G_1_ population. (E) Microtubule network disruption of Hep-J5 cells were after treating with 10 nM TCD. Cells were stained with an antibody against α-tubulin (red) and with DAPI (blue for nuclei).

Because TCD is a novel thiocolchicine derivative, we investigated the effect of TCD on cellular microtubule networks by immunofluorescence staining. Within 24 h of 10 nM TCD treatment, disruption of microtubule networks in Hep-J5 cells was apparent (Fig 1E). Microtubule disruptions increased in a time-dependent manner, with more severe effects observed at 48 and 72 h.

We next analyzed the effects of TCD on the cell cycle to clarify the mechanism of TCD-induced cytotoxicity, as microtubule depolymerization is commonly associated with cell cell-cycle arrest (Fig 1E). A flowcytometric analysis revealed an increase in the G_2_/M cell population in both Hep-J5 and Mahlavu cells after 24 h of TCD treatment (Fig 1A and 1B and S2 Table). When higher concentrations of TCD were applied, the G_2_/M cell population further increased, suggesting that TCD exhibited a dose-dependent effect. In addition, G_2_/M arrest in TCD-treated cells was also time-dependent. Upon administration of 10 nM TCD, significant G_2_/M accumulation was detected when the treatment time increased from 12 to 48 h (Fig 1C and 1D and S2 Table). The flowcytometric profile also revealed an increase in hypodiploid cells (the sub-G_1_ fraction), indicating that apoptosis had occurred.

### TCD induced the ER stress signaling cascade

**Fig 2:**
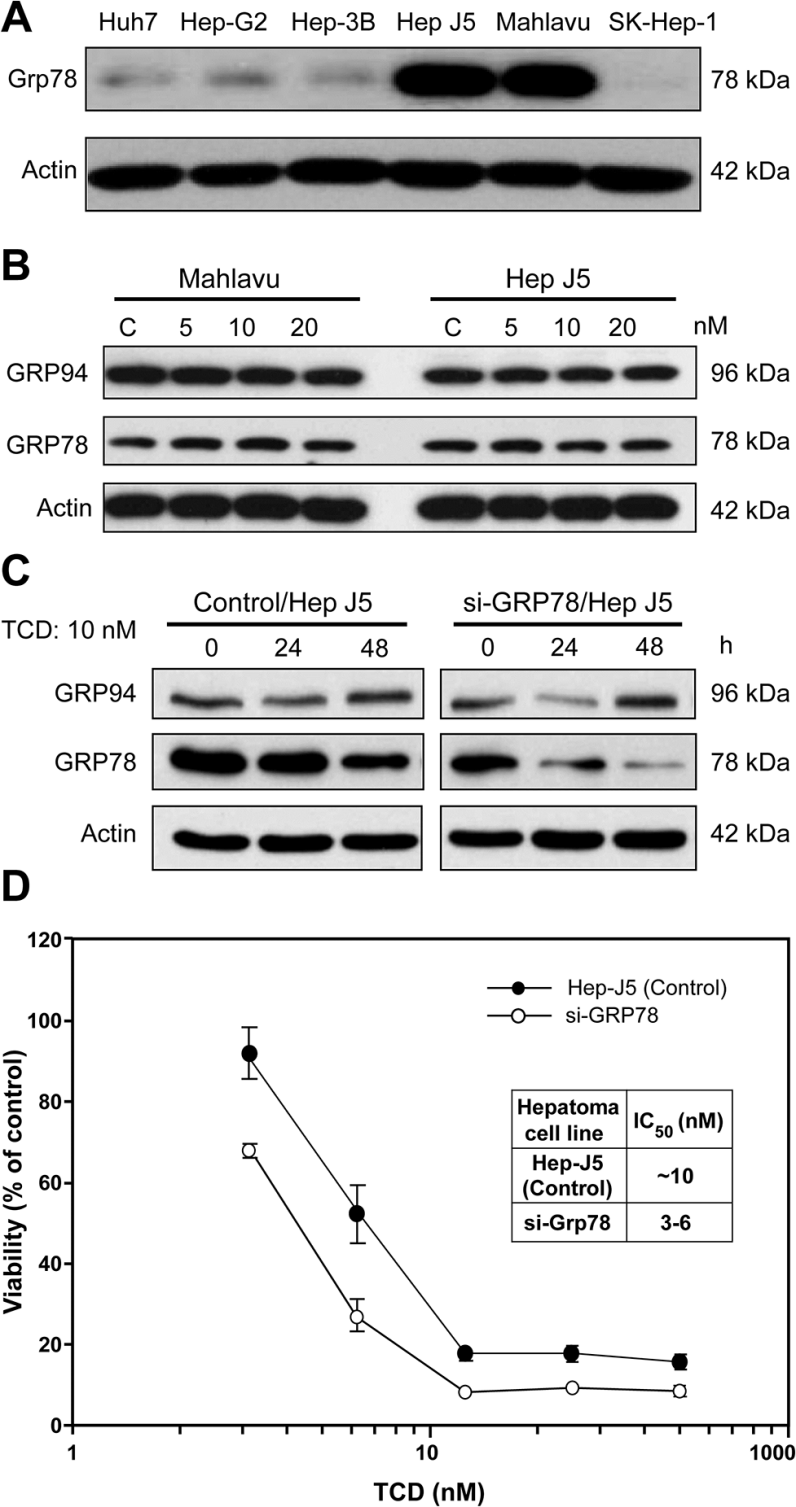
Expression of endoplasmic reticular (ER) chaperon proteins in TCD-treated Mahlavu and Hep-J5 cells. (A) Mahlavu and Hep-J5 cells are GRP78-overexpressing cell lines. (B) The dose effect and (C) time course of TCD treatment had no effect on GRP78 expression. GRP78 expression in HCC cells was assessed by western blotting. Forty micrograms of protein was loaded onto an 8% SDS-polyacrylamide gel. (D) Grp78 knockdown mildly enhanced the cytotoxic effects of TCD in Hep-J5 cells. Cells were seeded on 96-well plates at 5000 cells/well and treated with TCD for 72 h. Cell viability was detected using an SRB assay, and IC_50_ values were determined. Experiments were repeated at least two times, and results are expressed as the mean±SD.

**Fig 3:**
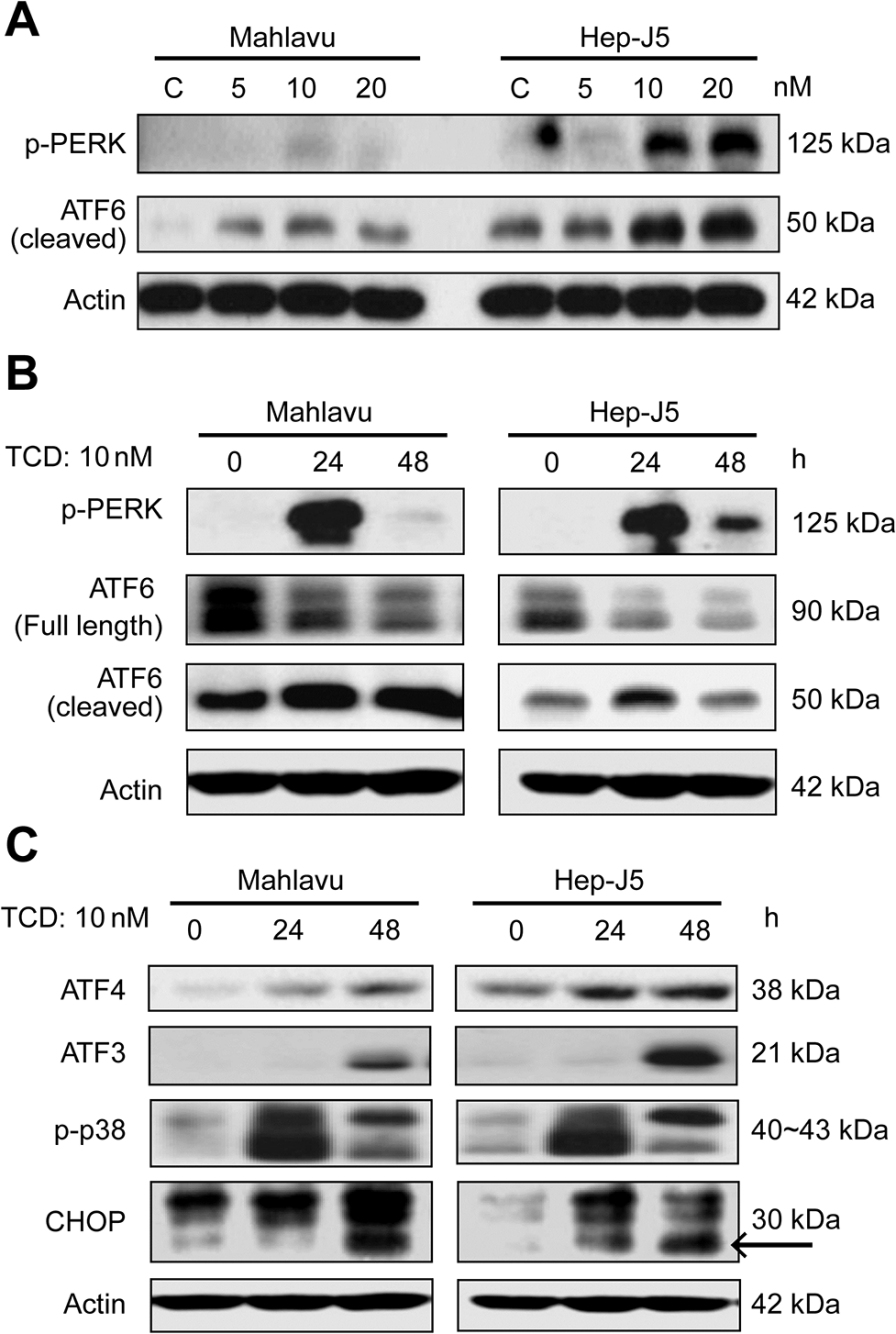
Dose effect and time course of activation of endoplasmic reticular (ER) stress effectors by TCD. (A) PERK phosphorylation and ATF6 cleavage increased in 10 or 20 nM TCD-treated groups. (B) This increased effect was also present in the 24 h TCD-treated group. (C) The downstream effectors, ATF4, ATF3, phospho-p38, and CHOP, showed increased expression in TCD-treated Mahlavu and Hep-J5 cells at 24 and 48 h. After treatment, protein expression in total protein extracts was assessed by western blotting. Experiments were repeated at least two times; a representative experiment is presented.

Mahlavu and Hep-J5cell lines both overexpress Grp78. Their high sensitivity to TCD treatment implied that the microtubule network disruption detected after TCD treatment might be associated with Grp78 expression (Fig 2A). However, TCD treatment did not affect the expression patterns of ER chaperone proteins, such as Grp94 and Grp78, in either a dose- or time-dependent manner (Fig 2B and 2C). Similarly, Grp78 knockdown mildly reduced the sensitivity of Hep-J5 cells to TCD (Fig 2D). Since Grp78 is a critical ER chaperone protein that mediates ER stress signals [28–30], we also investigated the effect of TCD on ER stress signaling. Interestingly, Grp78 downstream signaling factors, ATF6 and PERK, were activated after TCD treatment (Fig 3A and 3B), and these effects may lead to activation of ATF4, ATF3, p-p38, and CHOP (Fig 3C). These data implied that disruption of microtubule networks by TCD caused ER stress.

### TCD induced MMP loss, caspase activation, and apoptosis

**Fig 4:**
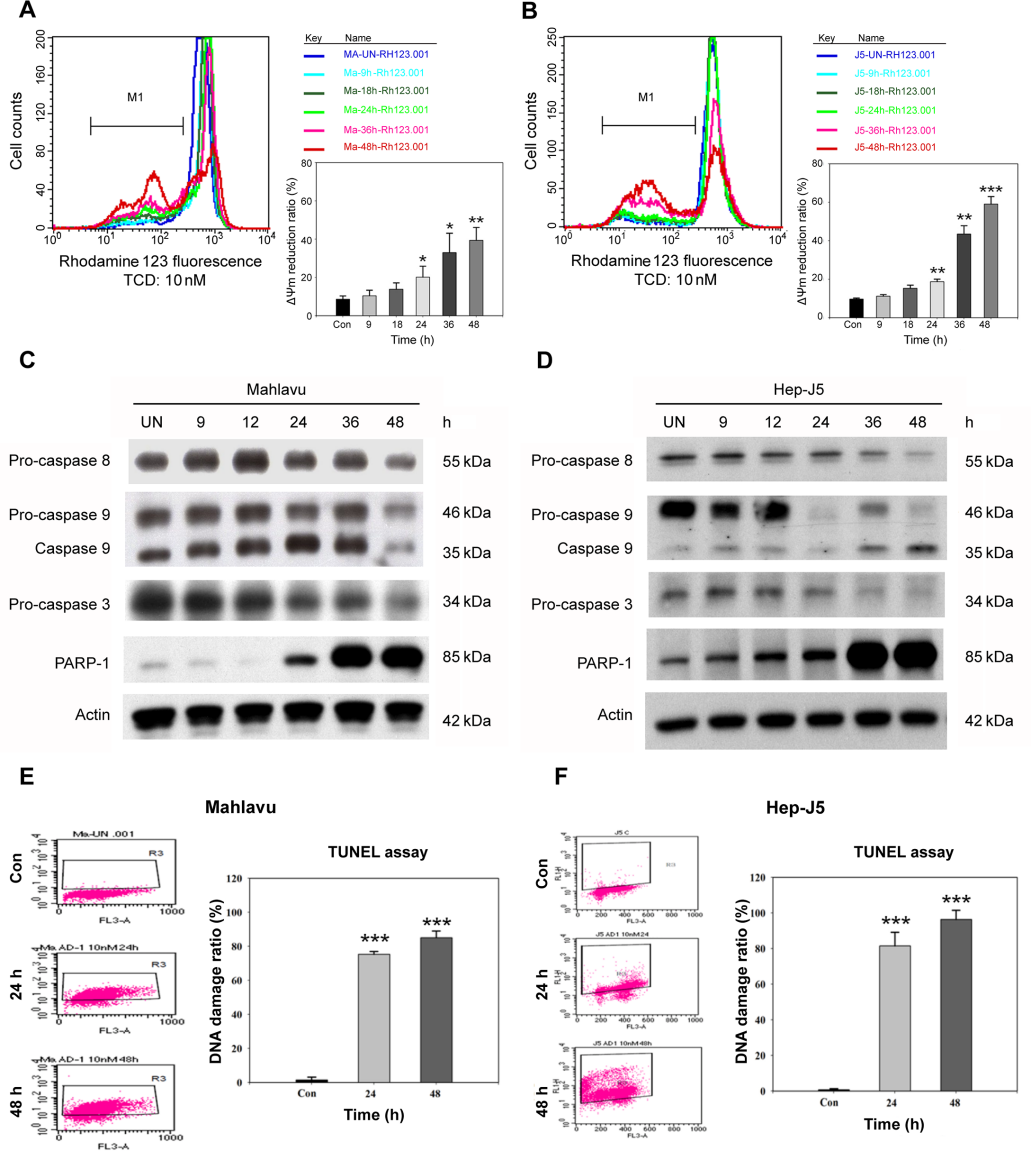
Reduction of the mitochondrial membrane potential (MMP) and activation of apoptotic proteins are involved in TCD-induced DNA fragmentation and apoptosis. Mahlavu (A) and Hep-J5 (B) cells showed increased rhodamine 123 fluorescence and reduced MMP in the 24–48 h TCD treatment group. Cells were treated with 10 nM TCD for different time periods. After incubation with TCD, cells were incubated with rhodamine 123 and analyzed by flow cytometry. The percentage reduction in the MMP is indicated. Apoptotic protein levels and DNA fragmentation were measured in cells. Mahlavu (C) and Hep-J5 cells (D) were treated with 10 nM TCD for the indicated times and procaspase-3, procaspase-8, procaspase-9, and cleaved PARP expression levels were analyzed. A TUNEL analysis of Mahlavu (E) and Hep-J5 (F) cells showed increased DNA fragmentation in apoptotic cells after treatment with 10 nM TCD for 24 and 48 h. The percent DNA damage increased over the time course of TCD administration in both Mahlavu (E) and Hep-J5 (F) cells. Experiments were repeated at least two times, and results are expressed as the mean±SD (*p < 0.05; **p < 0.01; **p < 0.001).

Chemical-induced apoptosis is often associated with MMP collapse as well as caspase activation. We examined the effect of TCD on the MMP of HCC cell lines using rhodamine 123 labeling. As shown in Fig 4A and 4B, 10 nM of TCD induced a time-dependent MMP loss in both Mahlavu and Hep-J5 cells. After 48 h of treatment, ΔΨm was reduced 40% and 60% for Mahlavu and Hep-J5 cells, respectively.

In addition to MMP collapse, the cellular levels of apoptosis-associated caspase proteins also changed after TCD treatment. As shown in Fig 4C and 4D, procaspase-3, procaspase-8, and procaspase-9 proteins significantly decreased in both HCC cell lines after TCD treatment for 24 h. A subsequent analysis also detected an increase in the cleavage-form of PARP, a well-known substrate for caspase-3, between 12 and 48 h of TCD treatment.

PARP dysfunction could aggravate cellular DNA damage and promote apoptotic DNA fragmentation. Therefore, we used the TUNEL assay to evaluate DNA damage. Consistent with the PARP result, TCD had a time-dependent effect on cellular DNA damage (Fig 4E and 4F). After 24 h drug treatment, dead cells gradually increased in the TCD-treated group compared to controls. These results suggested that TCD could inhibit cancer cell growth via arresting cells in the G_2_/M phase, followed by activation of the apoptotic pathway.

### TCD exhibits potent antitumor activity against HCC cells in vivo

**Fig 5:**
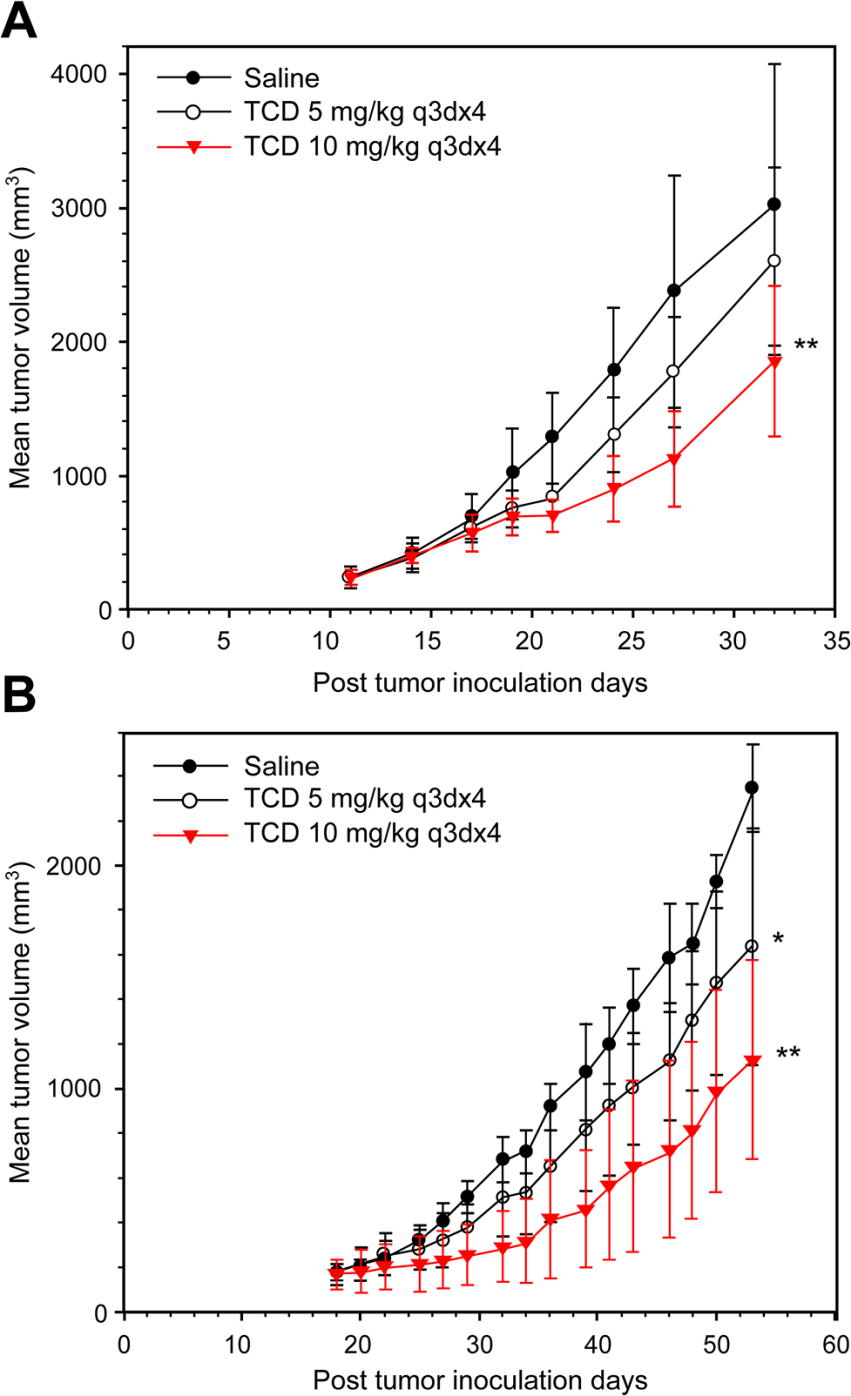
Therapeutic efficacy of formulated TCD against (A) Hep-J5 and (B) Mahlavu human hepatoma xenografts. Hepatoma cells were subcutaneously injected into mice. Mice were treated with saline, or 5 or 10 mg/kg TCD when the mean tumor diameter reached 5 mm. The difference in the mean tumor volume was statistically significant between saline- and TCD drug-treated mice (*p<0.05; **p<0.01).

To examine the therapeutic efficacy of TCD on hepatomas, we used Hep-J5 and Mahlavu cells in a xenograft tumor model (Fig 5A and 5B). TCD treatment resulted in marked tumor inhibition in both animal models. The percent change in Hep-J5 tumor volume for treated vs. control groups (% T/C) at the end point of the study (day 32) was 77.5% and 53% for 5 and 10 mg/kg TCD treatments, respectively (S2 Table). The efficacy of the 10 mg/kg over that of the 5 mg/kg dose in this animal study was also demonstrated in the Mahlavu model, where the % T/C values at the endpoint of the study (day 53) were 68.9% and 50.1% for 5 and 10 mg/kg, respectively (S2 Table).

## Discussion

Microtubules are a well-established drug target for treating rapidly dividing cancer cells, for which microtubule-targeting agents (MTAs) interfere with the dynamics of spindle microtubules during mitotic progression. Previous studies reported the use of anti-microtubule agents as a therapeutic strategy for HCC treatment [30, 31]. However, similar to most chemotherapeutic agents, MTAs show limited response and survival benefits in HCC patients. Here, we report that a new colchicine derivative, TCD, exhibited highly potent antitumor activity. Like most MTAs, TCD treatment depolymerized microtubules and led to cell cycle arrest at the G_2_/M phase. Further, TCD treatment contributed to induction of the caspase-dependent apoptotic pathway. In addition, this study showed that TCD triggered the ER stress-signaling pathway in both Mahlavu and Hep-J5 cells. Our finding is in accordance with several earlier reports that processes involving microtubule-based action and cytoskeletal organization are important biological functions in HCC [32, 33].

Colchicine is a natural antimitotic agent derived from a *Colchicum* alkaloid. Colchicine causes cell arrest in the mitotic phase by interfering with microtubule depolymerization both in vitro and in vivo [34]. However, the therapeutic effects of colchicine only occur at toxic and/or subtoxic dosages, which limits its clinical application [30, 35]. In this study, as a reference group, colchicine exhibited potent activity on different HCC cell lines. Compared to colchicine, TCD exhibited enhanced antitumor activity (IC_50_<50 nM) in different human HCC cell lines (Table 1), specifically the p53-null Hep-3B line, which is infected with the hepatitis B virus (HBV) [36], and the well-differentiated Huh7 cell line [37]. Although TCD showed increased antitumor activity in vitro, the in vivo toxicity of TCD is still a key issue regarding its clinical application. However, in a preliminary study, we found that the maximum tolerated doses of TCD and colchicine administered via intraperitoneal (IP) injections at day 40 (MTD_40_) were 150 and 1.6 mg/kg, respectively. Therefore, TCD has potential to be further developed as a therapeutic agent against HCC.

Previously, studies on the mechanisms underlying the effects of colchicines focused on its ability to block cell division by disrupting mitotic spindles, causing cell cycle arrest in the G_2_/M phase [38, 39]. As a colchicine derivative, TCD also disrupted microtubules and induced G_2_/M arrest in Hep-J5 cells **(** Fig 1A, 1B and 1E and S1 Table). Similarly, a low dosage of TCD (10 nM) induced drastic DNA damage, MMP loss, and an increased subG_1_ population in both Mahlavu and Hep-J5 cells (Fig 1B, 1C and Fig 4). Additionally, TCD presented a unique mechanism of action in this study where it was found to induce ER stress. TCD-induced ER stress minimized the protective effects of GRP78 and triggered activation of PERK and the ATF4-ATF3-CHOP axis (Fig 2 and Fig 3).

Interestingly, several reports show that inducers of ER stress caused synergistic effects with clinical drugs, such as MIRA-1 with doxorubicin [40] or vorinostat with sorafenib [41]. Specific clinical applications maybe realized using drug combinations [40, 41]. Moreover, cytochrome P450 (CYP) is a large protein family localized in ER membranes, and it plays a pathological role in liver disease and diabetes [42, 43]. Our results show that CHOP promotes mitochondrial dysfunction [44], and the TCD-mediated increase in CHOP expression was consistent with a reduction in the MMP (Figs 3C and 4A) and contributed to TCD-induced apoptosis.

Apoptotic agents are candidates for cancer therapy. Based on their site of action, extrinsic and intrinsic pathways are two mechanisms induced by anticancer agents [45]. Interaction with the death pathway triggers autoproteolytic cleavage of caspase-8, converting it to its active form, which then activates other procaspases, such as procaspase-3. Initiation of the intrinsic machinery results in the loss of MMP, which then activates caspase-9, an initiator caspase that leads to activation of caspase-3 [45, 46]. In the present data, we demonstrated that TCD induced both intrinsic and extrinsic apoptotic pathways in Hep-J5 and Mahlavu HCC cell lines (Fig 4B). Furthermore, proteolytic degradation of PARP was also involved in TCD-induced apoptosis (Fig 4B). Here, we suggest that caspase-3, caspase-8, and caspase-9 were activated in TCD-treated HCC cells.

**Fig 6:**
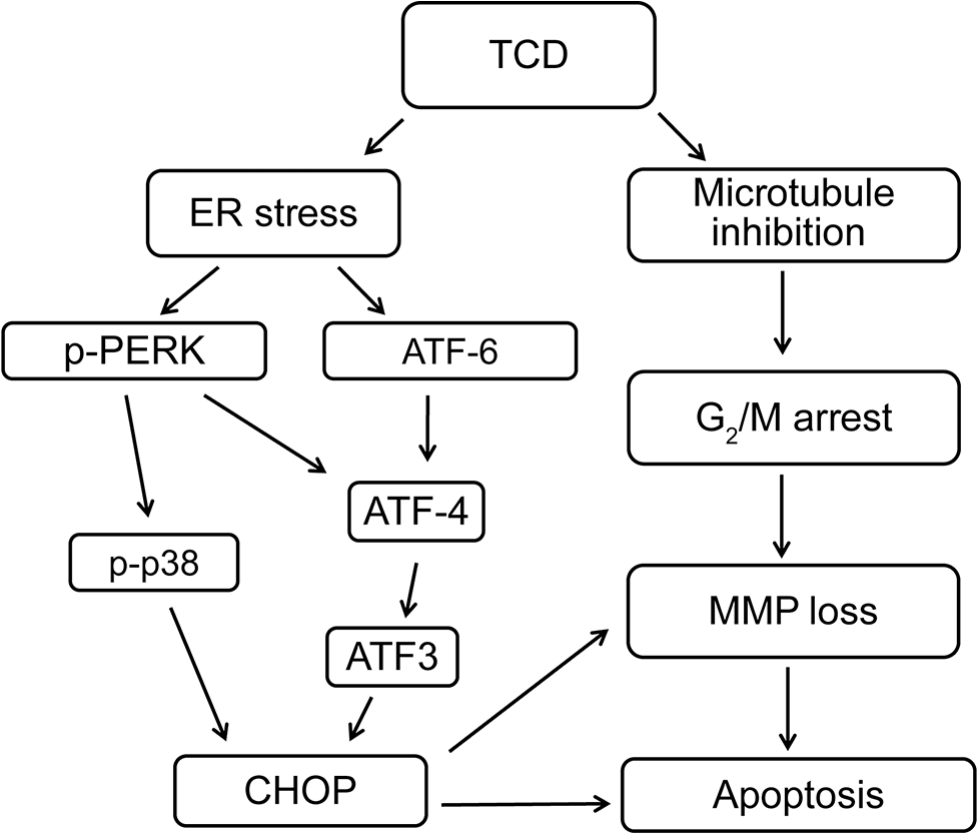
A diagrammatic presentation depicting the interplay of diverse mechanistic pathways involved in TCD-induced apoptosis of human hepatocellular carcinoma cells by both microtubule disruption and ER stress.

Microtubule-disrupting drugs, such as paclitaxel [47] and docetaxel [48], aggravate cell growth arrest and trigger apoptotic cell death in the G_2_/M phase. However, drug resistance occurs through stress-related mechanisms that prevent cell-cycle arrest. The mechanism of cell cycle modification through signal transduction, like that involving ROS/HIF-1 alpha [49], has been proven to increase cross-resistance to multiple antineoplastic agents. Our data showed that TCD had highly potent antitumor activity with a low IC_50_ level after treatment with 10 nM TCD in more highly malignant Hep-J5 and Mahlavu HCC cell lines [50] (Table 1, S2 Fig). Results of the cell-cycle progression experiment demonstrated that TCD effectively induced cell-cycle arrest at the G_2_/M phase after 12 h of treatment (Fig 1C and 1D and S1 Table). We also observed that TCD disrupted microtubule networks and triggered ER stress signaling in HCC cells in a time-dependent manner (Figs 1E, 3B and 3C). These results are summarized in Fig 6 and demonstrate the enhanced anticancer effects of TCD through a dual mechanism.

## Conclusions

Here, we demonstrated that TCD, a novel microtubule inhibitor, has potent antitumor activity in most human HCC cell lines. The anti-cancer activity of TCD is mediated by activation of both apoptotic and ER stress pathways (Fig 6). Using two HCC xenograft models, we further demonstrated that formulated TCD exhibits potent antitumor activity in vivo.

## Supporting Information

S1 Fig Chemical structures of TCD and colchicine. (TIF)

S2 Fig Cytotoxic effects of TCD and colchicine in HCC cell lines. (TIF)

S1 Table Effect of TCD on G_2_/M phase arrest and apoptosis. (PPTX)

S2 Table Antitumor activity of formulated TCD in a human hepatoma xenograft model. (PPTX)
