# Supplementary figures and images for: A Novel Microtubule-Disrupting Agent Induces Endoplasmic Reticular Stress-Mediated Cell Death in Human Hepatocellular Carcinoma Cells
Source: PLoS One. 2015 Sep 10;10(9):e0136340. doi: 10.1371/journal.pone.0136340 (PMC4565632; doi:10.1371/journal.pone.0136340)

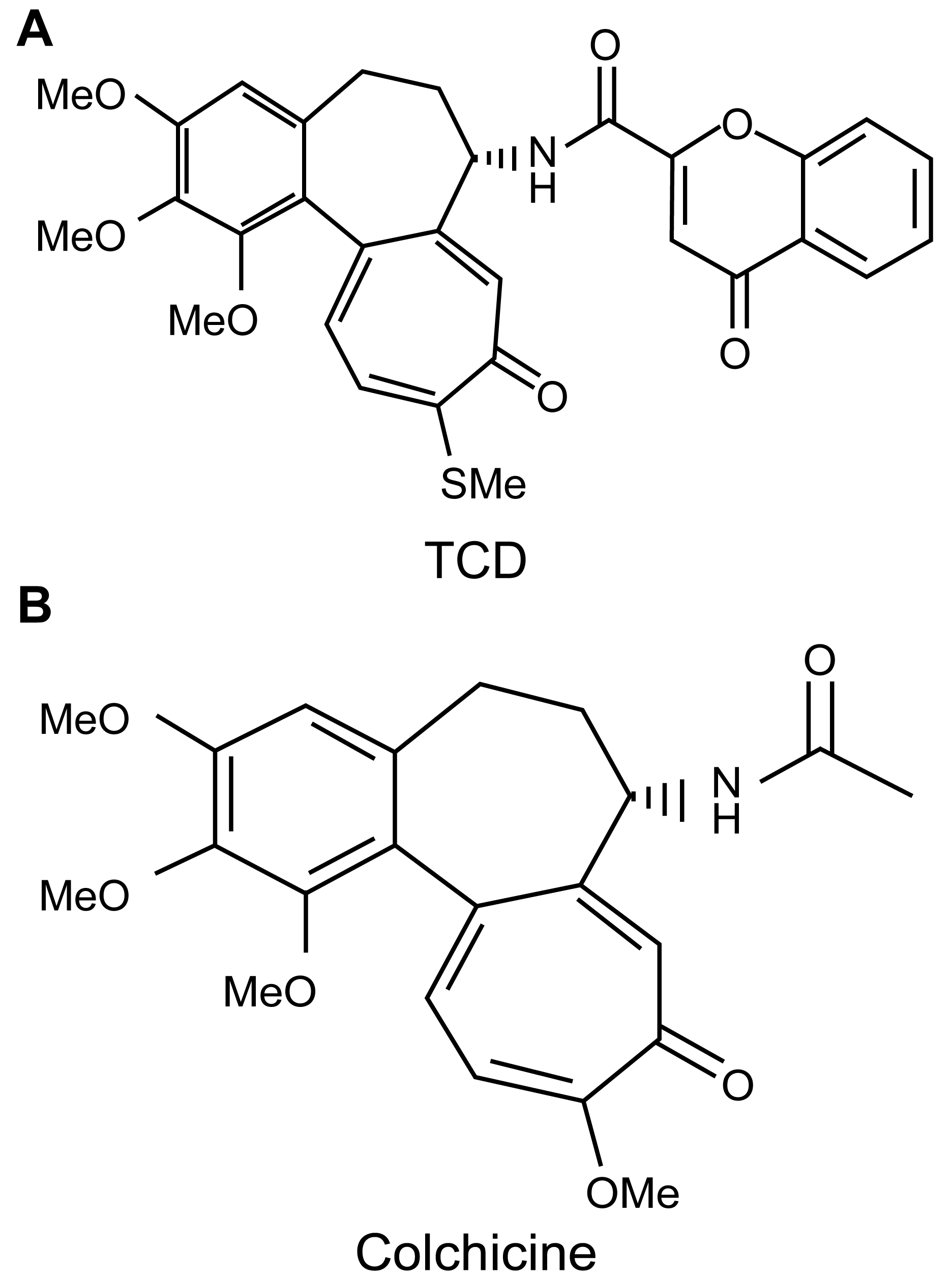

Supplement: S1 Fig — (TIF) [file pone.0136340.s001.tif]

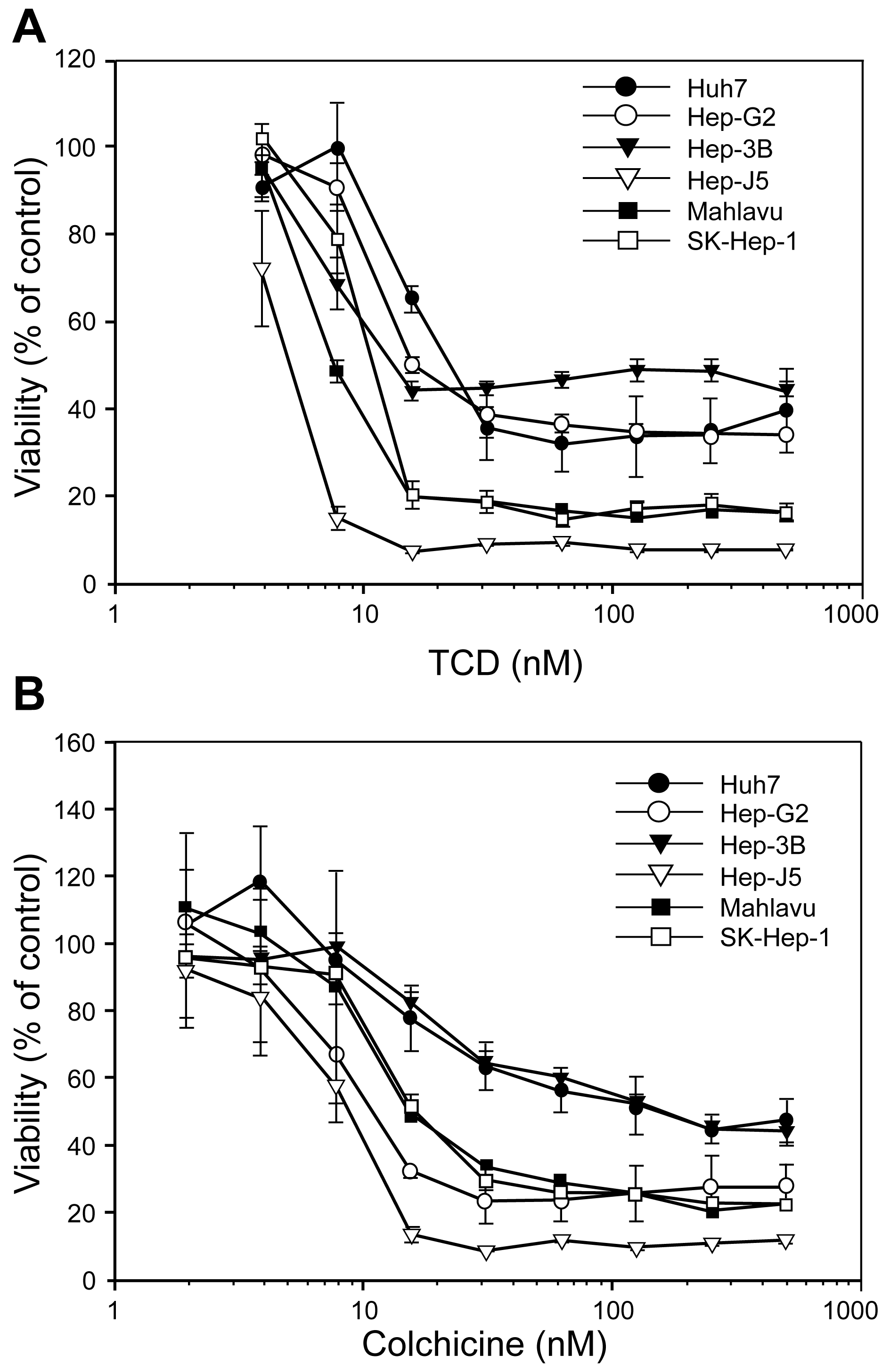

Supplement: S2 Fig — (TIF) [file pone.0136340.s002.tif]
