# Supplementary material for: A Novel Microtubule-Disrupting Agent Induces Endoplasmic Reticular Stress-Mediated Cell Death in Human Hepatocellular Carcinoma Cells
Source: PLoS One. 2015 Sep 10;10(9):e0136340. doi: 10.1371/journal.pone.0136340 (PMC4565632; doi:10.1371/journal.pone.0136340)
Supplement: S1 Table — (PPTX) [file pone.0136340.s003.pptx]

## Slide 1
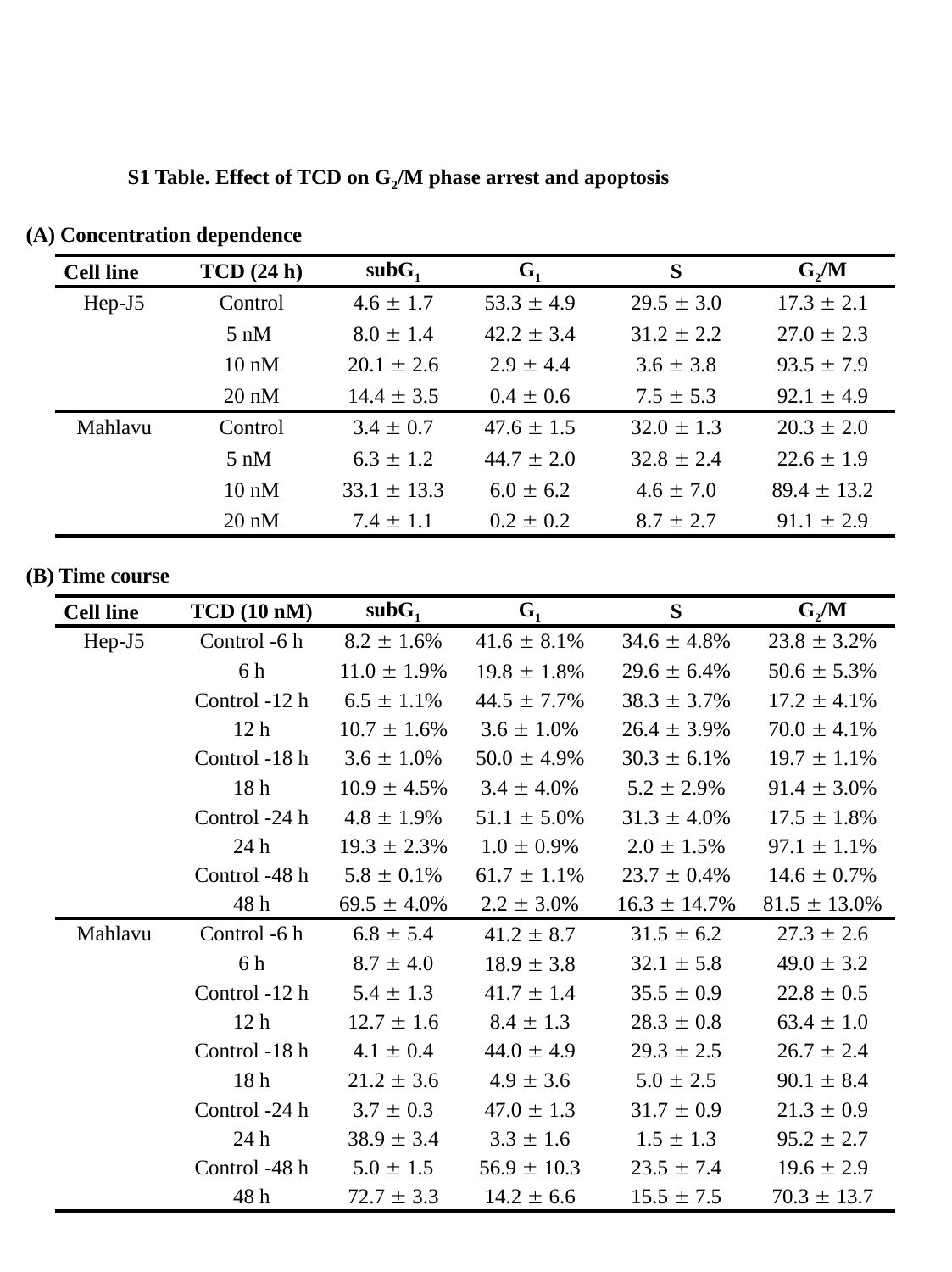

S1 Table. Effect of TCD on G2/M phase arrest and apoptosis
(A) Concentration dependence
| Cell line | TCD (24 h) | subG1 | G1 | S | G2/M |
| --- | --- | --- | --- | --- | --- |
| Hep-J5 | Control | 4.6 ± 1.7 | 53.3 ± 4.9 | 29.5 ± 3.0 | 17.3 ± 2.1 |
| | 5 nM | 8.0 ± 1.4 | 42.2 ± 3.4 | 31.2 ± 2.2 | 27.0 ± 2.3 |
| | 10 nM | 20.1 ± 2.6 | 2.9 ± 4.4 | 3.6 ± 3.8 | 93.5 ± 7.9 |
| | 20 nM | 14.4 ± 3.5 | 0.4 ± 0.6 | 7.5 ± 5.3 | 92.1 ± 4.9 |
| Mahlavu | Control | 3.4 ± 0.7 | 47.6 ± 1.5 | 32.0 ± 1.3 | 20.3 ± 2.0 |
| | 5 nM | 6.3 ± 1.2 | 44.7 ± 2.0 | 32.8 ± 2.4 | 22.6 ± 1.9 |
| | 10 nM | 33.1 ± 13.3 | 6.0 ± 6.2 | 4.6 ± 7.0 | 89.4 ± 13.2 |
| | 20 nM | 7.4 ± 1.1 | 0.2 ± 0.2 | 8.7 ± 2.7 | 91.1 ± 2.9 |
(B) Time course
| Cell line | TCD (10 nM) | subG1 | G1 | S | G2/M |
| --- | --- | --- | --- | --- | --- |
| Hep-J5 | Control -6 h | 8.2 ± 1.6% | 41.6 ± 8.1% | 34.6 ± 4.8% | 23.8 ± 3.2% |
| | 6 h | 11.0 ± 1.9% | 19.8 ± 1.8% | 29.6 ± 6.4% | 50.6 ± 5.3% |
| | Control -12 h | 6.5 ± 1.1% | 44.5 ± 7.7% | 38.3 ± 3.7% | 17.2 ± 4.1% |
| | 12 h | 10.7 ± 1.6% | 3.6 ± 1.0% | 26.4 ± 3.9% | 70.0 ± 4.1% |
| | Control -18 h | 3.6 ± 1.0% | 50.0 ± 4.9% | 30.3 ± 6.1% | 19.7 ± 1.1% |
| | 18 h | 10.9 ± 4.5% | 3.4 ± 4.0% | 5.2 ± 2.9% | 91.4 ± 3.0% |
| | Control -24 h | 4.8 ± 1.9% | 51.1 ± 5.0% | 31.3 ± 4.0% | 17.5 ± 1.8% |
| | 24 h | 19.3 ± 2.3% | 1.0 ± 0.9% | 2.0 ± 1.5% | 97.1 ± 1.1% |
| | Control -48 h | 5.8 ± 0.1% | 61.7 ± 1.1% | 23.7 ± 0.4% | 14.6 ± 0.7% |
| | 48 h | 69.5 ± 4.0% | 2.2 ± 3.0% | 16.3 ± 14.7% | 81.5 ± 13.0% |
| Mahlavu | Control -6 h | 6.8 ± 5.4 | 41.2 ± 8.7 | 31.5 ± 6.2 | 27.3 ± 2.6 |
| | 6 h | 8.7 ± 4.0 | 18.9 ± 3.8 | 32.1 ± 5.8 | 49.0 ± 3.2 |
| | Control -12 h | 5.4 ± 1.3 | 41.7 ± 1.4 | 35.5 ± 0.9 | 22.8 ± 0.5 |
| | 12 h | 12.7 ± 1.6 | 8.4 ± 1.3 | 28.3 ± 0.8 | 63.4 ± 1.0 |
| | Control -18 h | 4.1 ± 0.4 | 44.0 ± 4.9 | 29.3 ± 2.5 | 26.7 ± 2.4 |
| | 18 h | 21.2 ± 3.6 | 4.9 ± 3.6 | 5.0 ± 2.5 | 90.1 ± 8.4 |
| | Control -24 h | 3.7 ± 0.3 | 47.0 ± 1.3 | 31.7 ± 0.9 | 21.3 ± 0.9 |
| | 24 h | 38.9 ± 3.4 | 3.3 ± 1.6 | 1.5 ± 1.3 | 95.2 ± 2.7 |
| | Control -48 h | 5.0 ± 1.5 | 56.9 ± 10.3 | 23.5 ± 7.4 | 19.6 ± 2.9 |
| | 48 h | 72.7 ± 3.3 | 14.2 ± 6.6 | 15.5 ± 7.5 | 70.3 ± 13.7 |
