# Supplementary material for: A Novel Microtubule-Disrupting Agent Induces Endoplasmic Reticular Stress-Mediated Cell Death in Human Hepatocellular Carcinoma Cells
Source: PLoS One. 2015 Sep 10;10(9):e0136340. doi: 10.1371/journal.pone.0136340 (PMC4565632; doi:10.1371/journal.pone.0136340)
Supplement: S2 Table — (PPTX) [file pone.0136340.s004.pptx]

## Slide 1
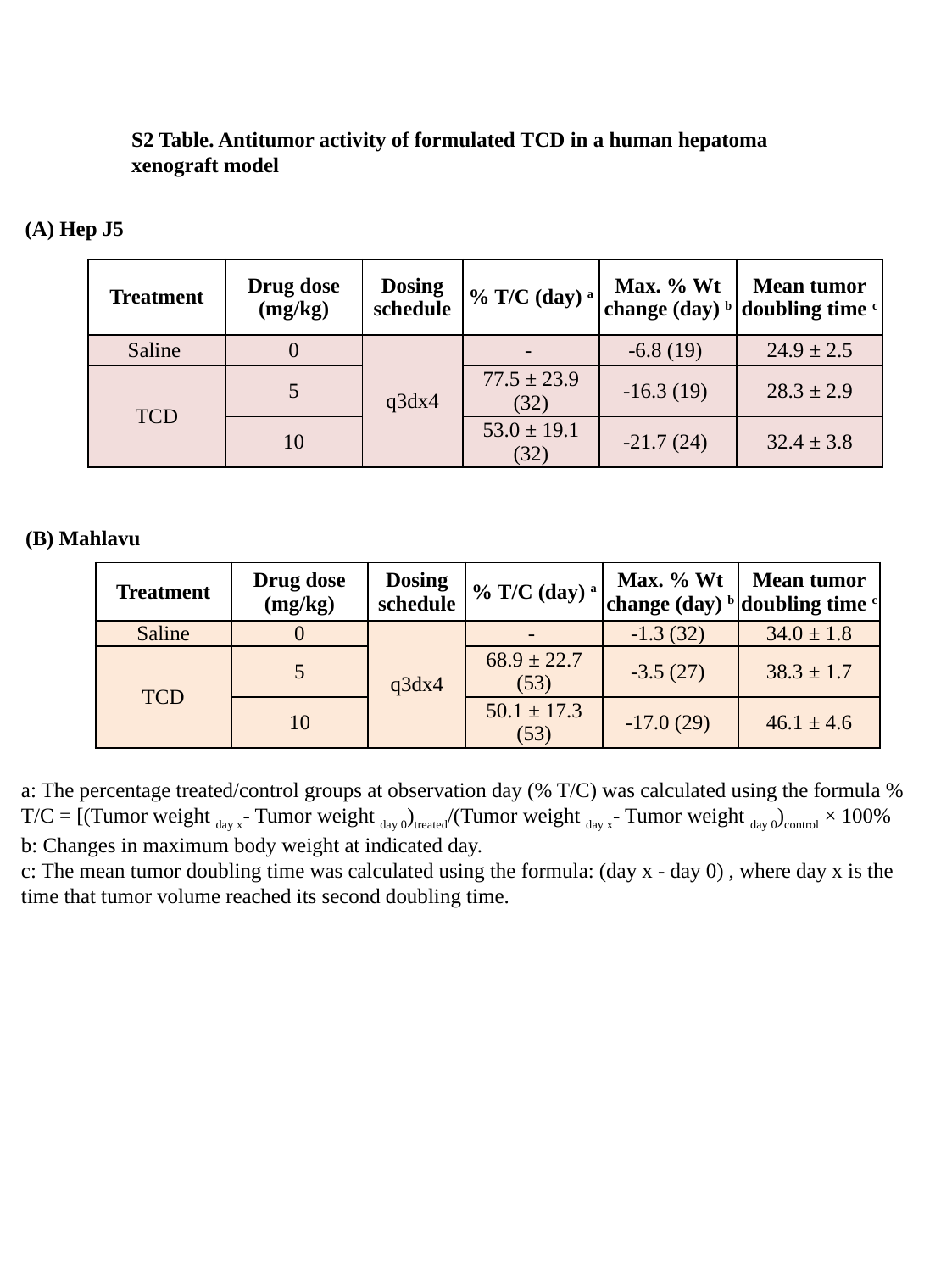

S2 Table. Antitumor activity of formulated TCD in a human hepatoma xenograft model
(A) Hep J5
| Treatment | Drug dose (mg/kg) | Dosing schedule | % T/C (day) a | Max. % Wt change (day) b | Mean tumor doubling time c |
| --- | --- | --- | --- | --- | --- |
| Saline | 0 | q3dx4 | - | -6.8 (19) | 24.9 ± 2.5 |
| TCD | 5 | | 77.5 ± 23.9 (32) | -16.3 (19) | 28.3 ± 2.9 |
| | 10 | | 53.0 ± 19.1 (32) | -21.7 (24) | 32.4 ± 3.8 |
(B) Mahlavu
| Treatment | Drug dose (mg/kg) | Dosing schedule | % T/C (day) a | Max. % Wt change (day) b | Mean tumor doubling time c |
| --- | --- | --- | --- | --- | --- |
| Saline | 0 | q3dx4 | - | -1.3 (32) | 34.0 ± 1.8 |
| TCD | 5 | | 68.9 ± 22.7 (53) | -3.5 (27) | 38.3 ± 1.7 |
| | 10 | | 50.1 ± 17.3 (53) | -17.0 (29) | 46.1 ± 4.6 |
a: The percentage treated/control groups at observation day (% T/C) was calculated using the formula % T/C = [(Tumor weight day x- Tumor weight day 0)treated/(Tumor weight day x- Tumor weight day 0)control × 100%
b: Changes in maximum body weight at indicated day.
c: The mean tumor doubling time was calculated using the formula: (day x - day 0) , where day x is the time that tumor volume reached its second doubling time.
